# Association of Ankle Brachial Index and Cognitive Function in Elderly Hypertensive Patients: A 2‐Year Longitudinal Study

**DOI:** 10.1002/brb3.71353

**Published:** 2026-03-29

**Authors:** Hui Chen, Min Li, Lei Liu, Anshi Shi, Zhe Yin, Gang Tian

**Affiliations:** ^1^ Hypertension Department, Central China Subcenter of National Center For Cardiovascular Diseases, Henan Cardiovascular Disease Center, Fuwai Central‐China Cardiovascular Hospital Central China Fuwai Hospital of Zhengzhou University Zhengzhou China; ^2^ Department of Cardiology The First Affiliated Hospital of Xi'an Jiao Tong University Xi'an China; ^3^ Department of Internal Medicine Hancheng Mining Bureau General Hospital Hancheng China

**Keywords:** ankle brachial index (ABI), cognitive function, elderly hypertension, Mini‐Mental State Examination (MMSE)

## Abstract

**Background:**

Ankle brachial index (ABI) is widely used to evaluate peripheral artery disease and atherosclerosis. Given the recognized links between atherosclerosis and cognitive impairment, this study was designed to evaluate the association between ABI and cognitive function in elderly hypertensive patients.

**Methods:**

A total of 408 elderly hypertensive patients from two hospitals were included and followed up for two years in 3 groups according to the ABI tertiles (Low ABI, ABI < 1.07 (n = 140); Intermediate ABI 1.07‐1.12 (n = 136), High ABI > 1.12 (n = 132)). Cognitive function was assessed by the Mini‐Mental State Examination (MMSE). Multiple analyses were used to evaluate the connection between ABI and cognitive function. Subgroup analyses along with interaction tests were done to see if this link was consistent. The analysis of covariance (ANCOVA) was used to compare the cognitive decline among 3 groups during a 2‐year follow‐up adjusted for related confounding factors.

**Results:**

ABI was independently correlated with MMSE score (B: 9.844, 95% CI: 7.088–12.600, *P*<0.001), while the blood pressure‐related parameters and brachial‐ankle pulse wave velocity (BaPWV) were not. The frequencies of cognitive impairment decreased among the 3 groups (22.1%, 8.1%, 3.0%, *P* < 0.001). Compared with high ABI group, low ABI group was associated with a higher prevalence of cognitive impairment (OR: 5.980, 95% CI: 1.966‐18.187, *p* = 0.015). ABI was negatively associated with cognitive impairment (OR: 0.057, 95% CI: 0.014‐0.787, *p* = 0.026), and this association was more pronounced in the old‐old subgroup. The MMSE score decline value decreased among 3 groups overall (F = 9.619, *P* < 0.001) and old‐old (F = 5.333, *p* = 0.006) hypertensive patients over a 2‐year follow‐up, but not in the young‐old patients (F = 2.345, *p* = 0.098).

**Conclusions:**

ABI is associated with cognitive impairment and cognitive function decline in elderly hypertensive patients, superior to the blood pressure‐related parameters and BaPWV. Besides, the association is stronger in the old‐old than in the young‐old.

## Graphical abstract

Our study was designed to evaluate the association between ABI and cognitive function in elderly hypertensive patients. Through a 2‐year follow‐up, we found that ABI is associated with cognitive impairment and cognitive function decline in elderly hypertensive patients, superior to the blood pressure‐related parameters and BaPWV. Besides, the association is stronger in old‐old than those in young‐old

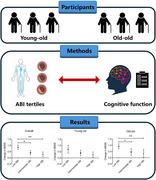

**Abbreviations**
ABI: ankle‐brachial index
ASCVD: arteriosclerotic cardiovascular disease
BaPWV: brachial‐ankle pulse wave velocity
BMI: body mass index
DBP: diastolic pressure
FBS: fast blood sugar
HDL‐C: high density lipoprotein cholesterol
HR: heart rate
LDL‐C: low density lipoprotein cholesterol
MMSE: Mini Mental State Examination
PI: pulse pressure index
PP: pulse pressure
SBP: systolic blood pressure
SCr: serum creatinine
TC: total cholesterol
TG: triglyceride

## 1 Introduction

With the aging of the population, the morbidity of cognitive impairments is increasing, which seriously aggravates the public healthcare burden. Reports showed that over 60% of the elderly had hypertension, and approximately 10 ∼ 45% of the elderly experienced cognitive impairment. (Ferri et al. 2005, Mills et al. 2016) Age and hypertension are both risk factors for vascular dementia and Alzheimer's disease. (Ninomiya et al. 2011, Shah et al. 2012) Therefore, the elderly hypertensive patients are at a high risk of cognitive impairment. Unfortunately, there is no curative treatment for cognitive impairment now, so identifying and mitigating the risk factors for cognitive impairment may be the most effective approach.

Generally, high blood pressure (BP) is believed to be associated with cognitive impairment. Previous studies revealed that the frequency of cognitive impairment increased in hypertension patients with higher BP (Muela et al. 2017), increased daytime BP variability was associated with poorer cognitive function (McDonald et al. 2017), and high nocturnal systolic blood pressure (SBP) was associated with lower MMSE scores in hypertensive patients (Yano et al. 2011). However, the BP type in elderly hypertensive patients is usually isolated systolic hypertension with high SBP and relatively low diastolic blood pressure (DBP). Therefore, the BP level may not be a stable and effective maker for evaluating cognitive function in the elderly. Morris et al. even found that high BP was a protective factor against cognitive impairment in older people. (Morris et al. 2002)

Ankle‐brachial index (ABI) is the ratio of ankle artery systolic pressure to brachial artery systolic pressure, which was originally proposed for the non‐invasive diagnosis of peripheral artery disease (PAD). (Alahdab et al. 2015, Xu et al. 2013) Pulse wave velocity (PWV) refers to the rate at which a pulse wave propagates along the artery wall from a specific location to another specific location. ABI and PWV, which could reflect arterial stenosis and arterial stiffness, respectively, are commonly used for the assessment of atherosclerosis. Given the close link between vascular disease and cognitive impairment, there have been population‐based studies on the association between ABI/PWV and cognitive function. Hajjar et al. (2016) found that higher PWV was superior to blood pressure in predicting cognitive decline in healthy adults. ABI was a good indicator for cognitive impairment in community health populations, patients with acute lacunar infarction, and those undergoing hemodialysis. (Buscemi et al. 2017, Tarraf et al. 2018, Huang et al. 2020, Wu et al. 2019) Besides, ABI has been confirmed to be associated with cognitive impairment in community‐dwelling older populations, superior to PWV (Sugawara et al. 2010). Therefore, atherosclerosis indicators, especially ABI, may be good predictors for cognitive impairment. However, the relationship between ABI and cognitive function in elderly hypertensive patients has rarely been reported.

In the current study, the elderly hypertensive patients were recruited from the First Affiliated Hospital of Xi'an Jiao Tong University and the Hancheng Mining Bureau General Hospital and followed up for two years. The aim of this study was to evaluate the association of ABI with cognitive function and cognitive function decline over 2 years in elderly hypertensive patients.

## 2 Methods

### 2.1 Study Design and Population

**FIGURE 1:**
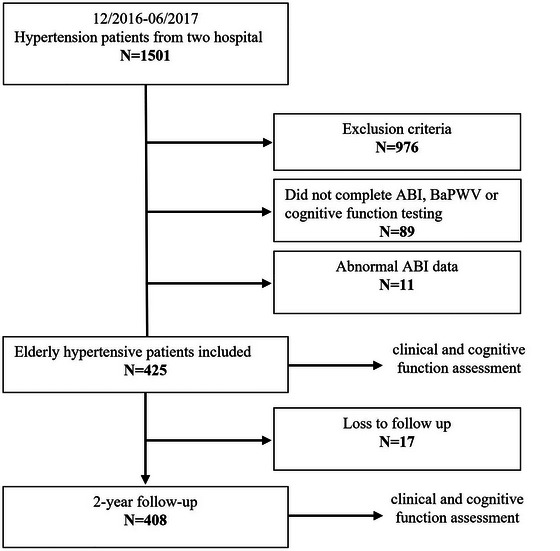
Flow chart of study recruitment and attrition. ABI, ankle‐brachial index.

A total of 1501 hypertension patients from the First Affiliated Hospital of Xi'an Jiao Tong University and the Hancheng Mining Bureau General Hospital were consecutively recruited from December 2016 to June 2017. In these patients, 976 of them were excluded according to the exclusion criteria, which were patients (1) aged < 60 years; (2) SBP ≥ 190 mmHg, or DBP < 60 mmHg; (3) secondary hypertension; (4) stroke, transient ischemic attack or neurologic deficits history; (5) hospitalization for acute myocardial infarction or unstable angina in the past six months; (6) revascularization in the past twelve months or future six months; (7) persistent atrial fibrillation; (8) hospitalization for heart failure in the past six months; (9) valvular heart disease or cardiomyopathy; (10) poorly controlled diabetes (fasting blood glucose (FBG) ≥ 11.1 mmol/L or hemoglobin A1c (HbA1c) > 8%); (11) malignant tumor, severe liver or kidney disease; or (12) refusal to sign the informed consent. Further, we excluded 89 patients who had incomplete data on the ABI, brachial‐ankle pulse wave velocity (BaPWV), or MMSE scores, 11 patients who had abnormal ABI data, and 17 patients who failed to follow up. At last, a total of 408 elderly hypertensive patients underwent clinical and cognitive function assessments at baseline and two years follow up were included in the final analysis (Figure 1). According to the average ABI tertile (Low ABI, ABI < 1.07 (n = 140); Intermediate ABI, 1.07–1.12 (n = 136), High ABI, ABI > 1.12 (n = 132)), the included patients were divided into 3 groups. The tertile approach was chosen based on previous studies that demonstrated a non‐linear relationship between ABI and cognitive outcomes, with risk varying across different ABI ranges rather than following a simple linear pattern. (Tarraf et al. 2018, Sugawara et al. 2010, Fowkes et al. 2008, Hanssen et al. 2012)

The study protocol was approved by the Ethical Committee of the First Affiliated Hospital of Xi'an Jiao Tong University, and the study conformed to the Declaration of Helsinki.

### 2.2 Measurement of Ankle‐Brachial Index (ABI) and Brachial‐Ankle Pulse Wave Velocity (baPWV)

The ABI was measured and calculated using a standard method. (Aboyans et al. 2012) The specific method was as follows. The SBP of the ankle artery and brachial artery were measured using a volume‐plethysmographic apparatus (VaSera VS‐1000, Fukuda Denshi Co, Tokyo, Japan). The ABI was calculated by dividing the value of the ankle artery systolic pressure value by the brachial artery systolic pressure value on each side, and the lower value was used for the analysis. The ABI values higher than 1.40 suggested poorly compressible leg arteries, which were excluded from the analysis. (Aboyans et al. 2012)

Brachial‐ankle pulse wave velocity (baPWV) was also measured by the volume‐plethysmographic apparatus (VaSera VS‐1000, Fukuda Denshi Co, Tokyo, Japan). The baPWV was calculated by the time‐phase analysis of brachial arteries and the volume waveforms at both ankles. The distance between the arm and ankle was estimated from the body height. The higher baPWV value was used for the analysis. BaPWV was reported to be significantly related to the aortic PWV. (Yamashina et al. 2002)

### 2.3 Assessment of Cognitive Function

Cognitive function was assessed face‐to‐face by specially trained physicians through the Mini‐Mental State Examination (MMSE), which included orientation, short‐term memory, ability to recall, calculation, ability to construct a sentence, and oral language ability. (Folstein et al. 1975) The MMSE score ranges from 0 to 30 points, and lower scores reflect a greater level of cognitive impairment. An MMSE score of < 24 is recognized as indicating cognitive impairment. (Mitchell 2009)

### 2.4 Definition of Age Stratification and Traditional Cardiovascular Risk Factors

According to the World Health Organization (WHO) age classification criteria for the elderly and the hypertension management guidelines, the elderly hypertensive patients were divided into the young‐old hypertensive patients (60 ∼ 74 years old) and the old‐old hypertensive patients (75 ∼ 89 years old). (Shimamoto et al. 2014, Baltes and Smith 2003, Li et al. 2021) Diabetes mellitus was defined as a diagnosis by physicians prior to admission or receiving hypoglycemic treatments or serum HbA1c% levels of ≥ 6.5%. Dyslipidemia was defined as a diagnosis by physicians prior to admission or a fasting serum total cholesterol (TC) level of > 5.7 mmol/L, low‐density lipoprotein cholesterol (LDL‐C) level of > 3.6 mmol/L, high‐density lipoprotein cholesterol (HDL‐C) level of < 0.7 mmol/L, triglyceride (TG) level of > 1.7 mmol/L, or the intake of lipid‐lowering medications. Smoking was defined as “yes” or “no” according to self‐reported information. Body mass index (BMI) was calculated as body weight (kg)/ height (m)^2^. BP was measured using a mercury sphygmomanometer. After resting for five minutes in a sitting position, the right upper limb BP of the patients was measured twice at least one minute apart. SBP and DBP were recorded as phase I and V Korotkoff sounds. If the difference between the two measurements exceeded 5 mmHg (1 mmHg = 0.133 kPa), the BP was measured again. The average BP was then recorded. Pulse pressure (PP) was calculated as SBP (mmHg)—DBP (mmHg). The pulse pressure index (PI) was calculated as PP (mmHg) / SBP (mmHg).

### 2.5 Measurement of Blood Parameters

Venous blood samples of all patients were drawn from the upper limb. The blood parameters were measured by the clinical laboratory of the First Affiliated Hospital of Xi'an Jiao Tong University. The patients were instructed to fast at least for 12 h before biochemical indicator investigations. The biochemical indicators included total cholesterol (TC), low density lipoprotein cholesterol (LDL‐C), high density lipoprotein cholesterol (HDL‐C), triglycerides (TG), serum creatinine (SCr), and fasting blood glucose (FBG).

### 2.6 Statistical Analysis

The continuous variables are presented as the mean ± SD or median (interquartile range), and the categorical variables are expressed as percentages. For the continuous variables, the Kolmogorov‐Smirnov test was applied to test the normality of the distribution, and one‐way ANOVA or the Kruskal‐Wallis test was used for the comparisons, depending upon the probability distribution of the variables. For the categorical variables, the chi‐squared test was used. Linear regression analysis was used to test the univariate correlation between the related variables and MMSE scores, and the significant correlation variables were used in further multiple linear regression analysis. Binary logistic regression analysis was used to evaluate the associations between the ABI and cognitive impairment. Furthermore, stratified analyses were conducted between different types of gender (male and female), age (young‐old (60–74y) and old‐old (75–89y), education (illiteracy or primary school, middle school, and high school or higher), diabetes, dyslipidemia, and ASCVD history in the subgroup patients. Cognitive function change was calculated as the difference between the baseline and follow‐up MMSE scores and compared by a paired‐samples t‐test. The cognitive decline differences among ABI tertiles were assessed by one‐way ANOVA, and further analyzed by analysis of covariance (ANCOVA) adjusted for age, gender, education, arteriosclerotic cardiovascular disease (ASCVD) history, and baseline MMSE scores. The estimated marginal means (with 95% confidence interval) from the models were calculated. Statistical analyses were performed using SAS software version 9.4 (SAS Institute, Inc., Cary, North Carolina, USA). All probabilities were 2‐sided and *p* value < 0.05 were considered statistically significant.

## 3 Results

### 3.1 Baseline Characteristics of Elderly Hypertensive Patients Across ABI Tertiles

The mean age of the entire study population (n = 408) was 72.93 ± 8.56 years, and 49.3% are males (Table 1). There was a decrease in age from low ABI group to high ABI group (*p* < 0.001). The patients in low ABI group had higher frequency of ASCVD history (*p* < 0.05). There was a significant increase in the MMSE score (25.54 ± 2.53, 26.79 ± 2.01, 24.71 ± 1.71; *p* < 0.001) and a decrease in the PP, PI, and baPWV from low ABI group to high ABI group (*p* < 0.01). However, there was no significant difference in other traditional cardiovascular risk factors (diabetes, dyslipidemia, smoking, and exercise), education, BMI, blood pressure, blood lipid levels, blood glucose, and other biochemical indicators across the three groups (*p* > 0.05). The use frequency of using antihypertensive drugs, antidiabetic drugs, antiplatelet drugs, and statins did not differ between the three groups (*p* > 0.05). Further, we perform analyses using MMSE sub‐scores. We found that there was a significant increase in the attention and calculation and delayed recall sub‐scores from low ABI group to high ABI group (*p* < 0.05) (Table S1).

**TABLE 1:** Baseline characteristics of elderly hypertensive patients across the ABI tertiles.

Variable	Overall (n = 408)	Low ABI (n = 140)	Intermediate ABI (n = 136)	High ABI (n = 132)	*P* for trend
Age (years)	72.93 ± 8.56	76.04 ± 8.07	72.47 ± 8.68	70.11 ± 8.56	0.000
Male, n (%)	201 (49.3)	68 (48.6)	62 (45.6)	71 (53.8)	0.398
Education (years)	7.21 ± 3.27	6.99 ± 3.36	7.32 ± 3.01	7.32 ± 3.45	0.774
Diabetes, n (%)	64 (15.7)	28 (20.0)	17 (12.5)	19 (14.4)	0.204
Dyslipidemia, n (%)	210 (51.5)	78 (55.7)	73 (53.7)	59 (44.7)	0.157
ASCVD history, n (%)	73 (17.9)	34 (24.3)	24 (17.6)	15 (11.4)	0.021
Smoking	118 (28.9)	47 (33.6)	38 (27.9)	33 (25.0)	0.283
Exercise (hours/week)	7.0 (2.0−10.5)	6.0 (0.0−7.0)	7.0 (2.0−7.5)	7.0 (2.0−10.5)	0.053
BMI (kg/m^2^)	25.06 ± 3.48	24.88 ± 3.43	25.17 ± 3.78	25.12 ± 3.25	0.867
HR (bpm)	71.37 ± 6.13	72.12 ± 6.34	71.08 ± 6.05	70.92 ± 6.02	0.438
SBP (mmHg)	153.02 ± 13.43	154.08 ± 13.45	152.67 ± 12.89	152.27 ± 13.98	0.505
DBP (mmHg)	87.97 ± 8.98	87.06 ± 8.87	87.85 ± 8.21	89.05 ± 9.76	0.188
PP (mmHg)	65.06 ± 9.55	67.01 ± 9.24	64.82 ± 9.45	63.23 ± 9.67	0.004
PI	0.42 ± 0.04	0.43 ± 0.04	0.42 ± 0.04	0.41 ± 0.05	0.001
ABI	1.09 ± 0.07	1.01 ± 0.05	1.09 ± 0.01	1.16 ± 0.03	0.000
BaPWV (m/s)	18.03 ± 2.24	18.65 ± 2.17	17.75 ± 2.44	17.67 ± 2.29	0.001
TC (mmol/L)	4.88 ± 1.70	4.64 ± 1.48	4.83 ± 1.57	5.15 ± 1.98	0.178
LDL‐C (mmol/L)	2.63 ± 1.16	2.41 ± 1.10	2.65 ± 1.08	2.82 ± 1.26	0.100
HDL‐C (mmol/L)	1.28 ± 0.44	1.20 ± 0.35	1.26 ± 0.38	1.36 ± 0.55	0.073
TG (mmol/L)	1.87 ± 1.09	2.01 ± 1.24	1.79 ± 1.11	1.82 ± 0.90	0.401
FBS (mmol/L)	6.24 ± 1.30	6.25 ± 1.14	6.03 ± 1.24	6.44 ± 1.46	0.175
SCr (µmol/L)	70.75 ± 13.86	68.10 ± 14.84	71.32 ± 13.55	73.11 ± 13.55	0.103
MMSE score	26.58 ± 2.26	25.54 ± 2.53	26.79 ± 2.01	27.47 ± 1.71	0.000
Antihypertensive drugs	336 (82.4)	112 (80.0)	110 (80.9)	114 (86.4)	0.923
Diuretics, n (%)	41 (10.0)	15 (10.7)	15 (11.0)	11 (8.3)	0.793
CCB, n (%)	222 (54.4)	73 (52.1)	70 (51.5)	79 (59.8)	0.763
ACEI/ARB, n (%)	158 (38.7)	51 (36.4)	48 (35.3)	59 (44.7)	0.639
Beta‐blocker, n (%)	55 (13.5)	18 (12.9)	20 (14.7)	17 (12.9)	0.876
Antidiabetic drugs, n (%)	46 (11.3)	22 (15.7)	7 (5.1)	17 (12.9)	0.103
Antiplatelet drugs, n (%)	62 (15.2)	20 (14.3)	27 (19.9)	15 (11.4)	0.265
Stain, n (%)	97 (23.8)	29 (20.7)	37 (27.2)	31 (23.5)	0.734

Low ABI, ABI < 1.07 (n = 140); Intermediate ABI 1.07‐1.12 (n = 136), High ABI > 1.12 (n = 132); MMSE, Mini Mental State Examination; ASCVD, arteriosclerotic cardiovascular disease; BMI, body mass index; HR, heart rate; SBP, systolic blood pressure; DBP, diastolic pressure; PP, pulse pressure; PI, pulse pressure index; ABI, ankle‐brachial index; BaPWV, brachial‐ankle pulse wave velocity; TC, total cholesterol; LDL‐C, low density lipoprotein cholesterol; HDL‐C, high density lipoprotein cholesterol; TG, triglyceride; FBS, fast blood sugar; SCr, serum creatinine; CCB, calcium channel blocker; ACEI, angiotensin converting enzyme inhibitor; ARB, angiotensin receptor blocker.

### 3.2 Correlation Between ABI and MMSE Scores

The univariate correlation analysis showed that age, education, ASCVD history, PP, PI, ABI, and baPWV were all significantly correlated with the MMSE scores, and the ABI was strongly correlated with MMSE scores (r = 0.380, *p* < 0.001) (Table S2). All the above significant correlation variables were taken into multiple linear regression analysis for further analysis. The results showed that age (B: −0.069, 95%CI: −0.093–−0.045, *p* < 0.001), education (B: 0.600, 95%CI: 0.148–1.053, *p* < 0.01), ASCVD history (B: −0.763, 95%CI: −1.260–−0.266, *p* < 0.01), and the ABI (B: 9.844, 95%CI: 7.088–12.600, *p* < 0.001) still had a strong significant correlation with MMSE scores, whereas the baPWV and blood pressure related parameters (PP and PI) did not (Table 2).

**TABLE 2:** Independent association variables of MMSE scores in multivariate linear regression analysis.

Variable	Unstandardized coefficient		Standardized coefficient	95%CI for B	*P*
	B	SEM	β		
Constant	20.868	2.368		16.212 to 25.523	0.000
Age	−0.069	0.012	−0.260	−0.093 to −0.045	0.000
Education	0.600	0.230	0.114	0.148 to 1.053	0.009
ASCVD history	−0.763	0.253	−0.129	−1.260 to −0.266	0.003
PP	−0.010	0.017	−0.043	−0.044 to 0.024	0.552
PI	1.838	3.882	0.035	−5.794 to 9.470	0.636
ABI	9.844	1.402	0.318	7.088 to 12.600	0.000
BaPWV	−0.006	0.042	−0.007	−0.088 to 0.075	0.878

MMSE, Mini Mental State Examination; ASCVD, arteriosclerotic cardiovascular disease; PP, pulse pressure; PI, pulse pressure index; ABI, ankle‐brachial index; BaPWV, brachial‐ankle pulse wave velocity; SEM, standard error of the mean; Adjusted R^2^ for this model = 0.284; *P* for this model < 0.001.

### 3.3 Association Between ABI and Cognitive Impairment and the Subgroup Analysis

**FIGURE 2:**
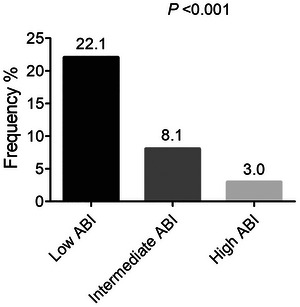
The frequency of cognitive impairment in elderly hypertensive patients based on the tertile of ABI. ABI, ankle‐brachial index.

An MMSE score of < 24 is recognized as indicating cognitive impairment. We found that the frequency of cognitive impairment in the overall patients was 11.3% (n = 46), and the frequency decreased from low ABI group to high ABI group (22.1% (n = 31), 8.1% (n = 11), 3.0% (n = 4), *p* < 0.001) (Figure 2).

The binary logistic regression analysis was used to analyze the association between ABI and cognitive impairment (Table 3). When the ABI was used as a continuous variable, it was significantly associated with the prevalence of cognitive impairment (OR: 0.057, 95% CI: 0.014–0.787, *p* = 0.026). Compared with the High ABI group, Low ABI group was significantly associated with a higher prevalence of cognitive impairment (OR: 5.980, 95% CI: 1.966–18.187, *p* = 0.015).

**TABLE 3:** Binary logistic regression analysis on the relationship of ABI and cognitive impairment.

	Continuous	*P*	Tertiles of the ABI			*P* for trend
			Low	Intermediate	High	
Model 1	0.029 (0.022−0.710)	0.009	9.101 (3.115−26.590)	2.816 (0.873−.079)	Ref	<0.001
Model 2	0.032 (0.018−0.737)	0.012	6.486 (2.169−19.391)	2.375 (0.727−7.754)	Ref	<0.001
Model 3	0.057 (0.014−0.787)	0.026	5.980 (1.966−18.187)	2.104 (0.634−6.981)	Ref	0.015

ABI, ankle‐brachial index;.

Model 1: Unadjusted;.

Model 2: Adjusted for age, sex, education, smoking, exercise;.

Model 3: Adjusted for age, sex, education, smoking, exercise, BMI, SBP, DBP, PP, PI, BaPWV and ASCVD history.

Table 4 showed the subgroup analysis for the association between ABI and cognitive impairment. When stratified by age, gender, education, and traditional cardiovascular risk factors, this association were significant in old‐old group (OR: 0.017, 95% CI: 0.005–0.095, *p* = 0.006), men (OR: 0.042, 95% CI: 0.012–0.873, *p* = 0.031), high school or higher (OR: 0.047, 95% CI: 0.011–0.521, *p* = 0.032), diabetes (OR: 0.045, 95% CI: 0.009–0.273, *p* = 0.102), and ASCVD history (OR: 0.029, 95% CI: 0.006–0.167, *p* = 0.027) patients. The difference of the association remained significant between different age groups (*P* for interaction, 0.035), while this difference was insignificant between other subgroups.

**TABLE 4:** Subgroup analysis for the association of ABI and cognitive impairment.

Subgroup	Total	MMSE score < 24, n (%)	OR	95% CI	*P*	*P* for interaction
Age						0.035
Young‐old (60‐74y)	236	18 (7.6)	0.173	0.079–1.175	0.113	
Old‐old (75‐89y)	172	28 (16.3)	0.017	0.005–0.095	0.006	
Gender						0.083
Male	201	21 (10.4)	0.042	0.012–0.873	0.031	
Female	207	25 (12.1)	0.113	0.058–2.181	0.192	
Education						0.098
Illiteracy or primary school	123	22 (17.9)	0.134	0.015–1.385	0.231	
Middle school	138	14 (10.1)	0.176	0.039–2.301	0.592	
High school or higher	147	10 (6.8)	0.047	0.011–0.521	0.032	
Diabetes						0.102
No	344	38 (11.0)	0.101	0.023–1.181	0.171	
Yes	64	8 (12.5)	0.045	0.009–0.273	0.023	
ASCVD history						
No	335	34 (10.1)	0.126	0.085–1.735	0.218	0.070
Yes	73	12 (16.4)	0.029	0.006–0.167	0.027	

MMSE, Mini Mental State Examination; All adjusted for age, education, diabetes, dyslipidemia, ASCVD history, smoking, PP, PI, and baPWV.

### 3.4 The Changes of Cognitive Function Among ABI Tertiles Over 2 Years

The overall mean MMSE score decline value (△MMSE) over 2 years in the low, intermediate, and high ABI groups was 0.78 ± 1.19, 0.43 ± 1.07, and 0.36 ± 0.92 points (all *p* < 0.001), respectively (Table 5). As the above results showed the association of ABI and cognitive impairment was different between young‐old and old‐old hypertensive patients, we also analyzed the changes of cognitive function in different age stratification patients. The MMSE scores of different ABI groups all declined during the 2‐year follow‐up in both young‐old and old‐old hypertensive patients (all *p* < 0.01). We further analyzed the difference of △MMSE among ABI tertiles by one‐way ANOVA. The △MMSE decreased among ABI tertiles in overall (△*p* = 0.002) and old‐old (△*p* = 0.010) patients, but not in young‐old (△*p* = 0.407) patients. (Table 5)

**TABLE 5:** The changes of cognitive function over 2 years among ABI tertiles.

	Low ABI (n = 140)					Intermediate ABI (n = 136)					High ABI (n = 132)				△*P*
	Baseline	2 years	△MMSE	*P*_1_		Baseline	2 years	△MMSE	*P*_2_		Baseline	2 years	△MMSE	*P*_3_	
Overall (n = 408)	25.54 ± 2.53	24.76 ± 2.76	0.78 ± 1.19	0.000		26.79 ± 2.01	26.35 ± 1.99	0.43 ± 1.07	0.000		27.47 ± 1.71	27.11 ± 1.77	0.36 ± 0.92	0.000	0.002
Young‐old (n = 236)	26.26 ± 2.34	25.74 ± 2.55	0.51 ± 1.14	0.005		27.41 ± 1.98	27.01 ± 1.81	0.40 ± 1.10	0.003		27.92 ± 1.58	27.58 ± 1.57	0.35 ± 0.99	0.001	0.407
Old‐old (n = 172)	25.22 ± 2.56	24.32 ± 2.74	0.90 ± 1.20	0.000		26.06 ± 1.80	25.59 ± 1.93	0.48 ± 1.03	0.001		26.43 ± 1.56	26.05 ± 1.75	0.38 ± 0.71	0.002	0.010

Low ABI, ABI < 1.07 (n = 140); Intermediate ABI 1.07‐1.12 (n = 136), High ABI > 1.12 (n = 132); MMSE, Mini Mental State Examination; ABI, ankle‐brachial index; △MMSE, the difference of MMSE score between 2 years and baseline; *P*_1_, the differences of MMSE score at 2 years relative to baseline in Low ABI group was assessed by paired samples t‐test; *P*_2_, the differences of MMSE score at 2 years relative to baseline in Intermediate ABI group was assessed by paired samples t‐test; *P*_3_, the differences of MMSE score at 2 years relative to baseline in High ABI group was assessed by paired samples t‐test; △*P*, the differences of △MMSE among the ABI tertiles were assessed using one‐way ANOVA.

**FIGURE 3:**
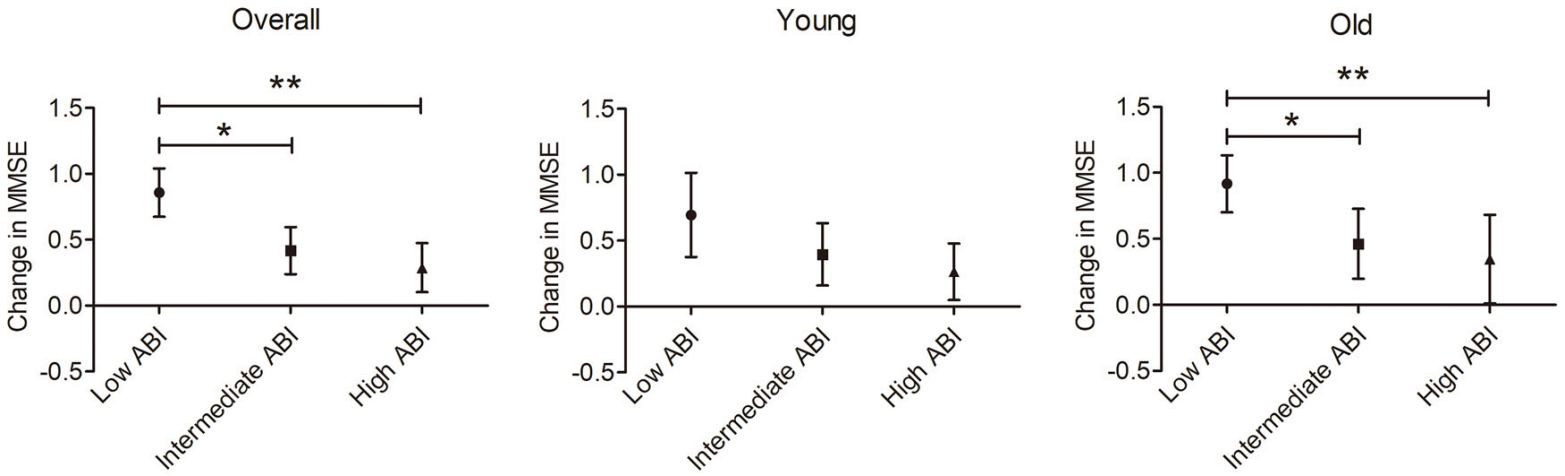
ANCOVA comparing cognitive decline according to the ABI tertiles. Low ABI, ABI < 1.07 (n = 140); intermediate ABI 1.07‐1.12 (n = 136), high ABI > 1.12 (n = 132); MMSE, Mini Mental State Examination; ABI, ankle‐brachial index; ANCOVA, analysis of covariance; all adjusted for age, gender, education, ASCVD history, and baseline MMSE scores.

To eliminate the influence of confounding factors, the ANCOVA was used to compare cognitive decline according to the ABI tertiles adjusted for age, gender, education, ASCVD history, and baseline MMSE scores. The low ABI group had significantly greater decline in MMSE score than those in the intermediate and high ABI groups overall (F = 9.619, *p* < 0.001) and old‐old (F = 5.333, *p* = 0.006) patients, but not in young‐old (F = 2.345, *p* = 0.098) patients. (Figure 3)

## 4 Discussion

The main findings of this study were that: 1, ABI had a stronger association with cognitive impairment in elderly hypertensive patients than blood pressure‐related parameters (SBP, DBP, PP, and PI) and baPWV; 2, ABI was associated with cognitive impairment and cognitive function decline in elderly hypertensive patients, especially in the old‐old patients.

In this study, we strictly selected the elderly primary hypertension patients without severe heart, brain, kidney, and other diseases, which could help to eliminate the influence of confounding factors from other diseases and reduce the attrition rate. Besides, the patients who had incomplete data on ABI, BaPWV, or MMSE scores, abnormal ABI data, and loss to follow‐up were also excluded. These exclusion criteria were necessary to ensure the integrity of the core data, the accuracy of the measurements, and the reliability of the cognitive assessment.

About one billion people worldwide are suffering from hypertension, and the prevalence significantly increases with age. (Fryar et al. 2017) Hypertension increases the risk of vascular cognitive impairment, and the mechanisms are complex. Studies have shown that vascular remodeling induced by long‐term hypertension increased brain microvascular resistance, damaged microvascular structure, decreased brain tissue blood perfusion, promoted endothelial dysfunction, and diminished cerebrovascular reactivity (Girouard and Iadecola 2006, Capone et al. 2012). It has also been shown to increase oxidative stress, inflammation, and vasoactive substances in brain tissue, leading to neuronal loss, and white matter degradation (Pires et al. 2013, De Silva and Faraci 2016, Ungvari et al. 2021). The former and latter effects are all associated with cognitive decline. Hypertension and aging both have deleterious effects on cognitive function. Therefore, it is of great clinical significance to explore the risk factors for cognitive impairment in hypertensive patients, especially in elderly hypertensive patients.

The ABI, calculated by dividing the ankle artery systolic pressure value by the brachial artery systolic pressure value, can be used to evaluate PAD and generalized atherosclerosis, and indirectly reflect peripheral blood perfusion. PAD often accompanies cerebrovascular disease. It had been reported that 23.0% of patients with PAD had a stroke and a transient ischemic attack, and 8.5% of patients with a prior stroke and a transient ischemic attack had PAD. (Cacoub et al. 2009) We speculated that ABI may be associated with brain tissue blood perfusion and could be a good indicator of cognitive function in elderly hypertensive patients. Although many studies have reported the association between the ABI and cognitive function (Buscemi et al. 2017, Tarraf et al. 2018, Huang et al. 2020, Wu et al. 2019), few studies have focused on the relationship between the ABI and cognitive function in elderly hypertensive patients. Our cross‐sectional study found that the ABI was associated with cognitive impairment independent of age, gender, education, ASCVD history, blood pressure related parameters (PP and PI), and baPWV, and the cohort follow‐up study also confirmed that cognitive function declined more rapidly in patients with a low ABI, revealing an association between the ABI and cognitive function in elderly hypertensive patients.

Besides, we found low ABI was associated with the declines in attention and calculation and delayed recall in elderly hypertensive patients through MMSE sub‐scores analysis. It aligns with the Toledo Study of Healthy Aging, which reported significant associations between low ABI and memory decline. (Bareiro et al. 2024) The selective impairment of attention and calculation functions in the low ABI group supports the frontal‐subcortical pattern of vascular cognitive impairment. (Parfenov et al. 2019, Cheshire et al. 2025) Atherosclerosis induced by endothelial oxidative stress and inflammation could lead to brain‐microvascular dysfunction, which contributed to the genesis of vascular cognitive decline. (Parfenov et al. 2019) Therefore, vascular cognitive impairment might be the key pathophysiological mechanism in low ABI related cognitive impairment in elderly hypertensive patients.

Many studies have focused on the associations between BP levels and cognitive function, but little consensuses were reached. (Muela et al. 2017, Morris et al. 2002, Pandav et al. 2003) A close association between a high midlife BP level and late‐life cognitive decline has been widely accepted, whereas the associations between late‐life BP level and cognitive function are controversial. (Walker et al. 2017, Walker et al. 2019) This study found that ABI was significantly associated with cognitive impairment and cognitive decline in elderly hypertensive patients, while the blood pressure‐related parameters (SBP, DBP, PP, and PI) were not. Isolated systolic hypertension is the common BP type in elderly hypertensive patients with high systolic blood pressure (SBP) and relatively low diastolic blood pressure (DBP). Besides, SBP, DBP, PP, and PI can be affected by many factors (drugs, emotions, exercises, etc.), which are in a continuously fluctuating state, so they may have some limitations in evaluating vascular function in hypertension patients. Therefore, the blood pressure‐related parameters may not be good indicators for cognitive impairment in elderly hypertensive patients.

The BaPWV is the pulse wave velocity in the brachial‐to‐ankle artery. The BaPWV is an indicator reflecting vascular compliance and can be used as a predictor of hypertension‐related arterial stiffness. (Dumor et al. 2018) In this study, compared with ABI, the BaPWV was not independently correlated with cognitive function in elderly hypertensive patients. Similarly, a previous study of patients with lacunar infarction has also demonstrated that a lower ABI, but not a higher BaPWV, was associated with cognitive decline (Nakamori et al. 2022). Additionally, among patients with acute stroke, compared with ABI, BaPWV had a weaker predictive power for 3‐month outcomes (Matsushima et al. 2017). The BaPWV represents arterial stiffness, while ABI is indicative of arterial stenosis or obstruction, which indicates a progressed atherosclerosis stage. Therefore, ABI could reflect the blood perfusion of tissue more accurately. Studies have demonstrated that the ABI was an impressive predictor of ischemic stroke. (Lee et al. 2020, Hong et al. 2016) Besides, the elderly hypertensive patients might already generally have arterial stiffness, which might disturb the evaluation of the association between BaPWV and cognitive function. Based on our research results and literature reports, we speculate that the ABI might be a more direct and stable indicator reflecting brain tissue blood perfusion and cognitive function in elderly hypertensive patients, compared with BaPWV.

The subgroup analysis of this study showed that ABI had a closer association with cognitive impairment in old‐old hypertensive patients. There are various pathophysiological differences between young‐old and old‐old hypertensive patients, and the blood pressure management strategies also differ. The old‐old patients have longer times of exposure to vascular risk factors, and tend to have worse microvascular function and structure, dysregulation of cerebral blood flow, and shrinkage of gray and white matter. (Oschwald et al. 2019) Subsequent follow‐up studies had also confirmed that the cognitive function of old‐old hypertensive patients with low ABIs declined more rapidly compared to young‐old hypertensive patients. The results implied that the ABI was associated with cognitive impairment and cognitive function decline in elderly hypertensive patients, especially in old‐old hypertensive patients. The explanation could be that the old‐old patients tend to have been affected by atherosclerosis risks during a more protracted period. Besides, in the young‐old hypertensive patients, there was a marginal effect of the ABI on the cognitive function, and in the old‐old patients, this effect was significant. (Laukka et al. 2014) Therefore, we speculate that early initiation of anti‐atherosclerotic treatment might be helpful to prevent cognitive impairment in elderly hypertensive patients.

This study also demonstrated that diabetes was not a risk factor for cognitive impairment, which was inconsistent with the results of previous reports. (Koekkoek et al. 2015, Yuan and Wang 2017, Xue et al. 2019) The patients with poorly controlled diabetes were excluded from this study, so the results might indirectly reflect that well‐controlled diabetes was not a risk factor for cognitive impairment in elderly hypertensive patients.

However, there were some limitations to this study. First, although the participants were from two hospitals in China, the relatively small sample size and short follow‐up time may limit the conclusions. Second, the large number of exclusion criteria, while methodologically justified, may have introduced selection bias and limited the generalizability of our findings to the broader elderly hypertensive population. Third, only MMSE scores were used to evaluate cognitive function, and we did not adopt other various cognitive tests representing multiple cognitive domains and did not use brain imaging to detect cerebral small vessel diseases and brain white matter lesions. Lastly, we did not detect the parameters of blood pressure variability, and we could not evaluate the effects of blood pressure variability on cognitive function.

## 5 Conclusion

In conclusion, ABI has a closer association with cognitive impairment and cognitive function decline in elderly hypertensive patients compared to the blood pressure‐related parameters and BaPWV, and the association is stronger in the old‐old patients. Early monitoring of the ABI in hypertensive patients is necessary, and the individualized anti‐atherosclerotic therapy considering the ABI might help prevent cognitive impairment in elderly hypertensive patients more effectively. Further studies on this topic are needed to ascertain the benefits of early anti‐atherosclerotic treatment for the cognitive function of elderly hypertensive patients.

## Author Contributions

All authors participated in the design, interpretation of the studies and analysis of the data and review of the manuscript. Hui Chen, and Gang Tian were responsible for the study design, literature review, and drafting of the original manuscript. Hui Chen, Min Li, Lei Liu, Anshi Shi, and Zhe Yin were responsible for patient enrollment and follow‐up. Min Li and Lei Liu analyzed the data. Gang Tian revised the manuscript and approved the final version.

## Funding

This study was supported by the Nature Science Foundation of China (No. 81873513, 81600574, and 30871042), Key Projects of Shaanxi Science and Technology Research and Development Plan (No.2018ZDXM‐SF‐049), Shaanxi Science and Technology Research and Development Plan of International Science and Technology (No. 2012 kw‐40‐01 and 2014 JM2‐8145), Joint Construction Project of Henan Medical Science and Technology (No. LHGJ20220103), and Henan science and technology research project (No. 252102310254).

## Ethics Statement

The study plan was approved by the Ethical Committee of the First Affiliated Hospital of Xi'an Jiao Tong University. All participants gave their informed consent prior to their inclusion in the study.

## Supporting information




**Supplementary Table**: brb371353‐sup‐0001‐TableS1.docx

**Supplementary Table**: brb371353‐sup‐0002‐TableS2.docx

## Data Availability

The datasets used and analyzed during the current study are available from the corresponding author upon reasonable request.
