# Supplementary material for: Association of Ankle Brachial Index and Cognitive Function in Elderly Hypertensive Patients: A 2‐Year Longitudinal Study
Source: Brain Behav. 2026 Mar 29;16(4):e71353. doi: 10.1002/brb3.71353 (PMC13112016; doi:10.1002/brb3.71353)
Supplement: Supplementary file 1 — Supplementary Table: brb371353‐sup‐0001‐TableS1.docx [file BRB3-16-e71353-s001.docx]

**Table S1 MMSE sub-scores of elderly hypertensive patients across the ABI tertiles**

| Variable | Overall  (n=408) | Low ABI  (n=140) | Intermediate ABI  (n=136) | High ABI  (n=132) | *P* for trend |
| --- | --- | --- | --- | --- | --- |
| Orientation | 9.20±0.90 | 9.04±0.99 | 9.22±0.92 | 9.35±0.76 | 0.1182 |
| Immediate recall | 2.85±0.36 | 2.82±0.38 | 2.82±0.38 | 2.89±0.31 | 0.3923 |
| Attention and calculation | 4.17±0.71 | 4.00±0.74 | 4.16±0.68 | 4.33±0.68 | 0.0172 |
| Delayed recall | 2.12±0.69 | 1.96±0.67 | 2.12±0.68 | 2.28±0.66 | 0.0165 |
| Language | 8.28±0.80 | 8.23±0.88 | 8.28±0.81 | 8.32±0.68 | 0.8021 |
| Visuospatial construction | 0.69±0.46 | 0.63±0.48 | 0.76±0.43 | 0.68±0.47 | 0.2497 |

Low ABI, ABI<1.07 (n=140); Intermediate ABI 1.07-1.12 (n=136), High ABI>1.12 (n=132); MMSE, Mini Mental State Examination; ABI, ankle-brachial index
