# Supplementary material for: Association of Ankle Brachial Index and Cognitive Function in Elderly Hypertensive Patients: A 2‐Year Longitudinal Study
Source: Brain Behav. 2026 Mar 29;16(4):e71353. doi: 10.1002/brb3.71353 (PMC13112016; doi:10.1002/brb3.71353)
Supplement: Supplementary file 2 — Supplementary Table: brb371353‐sup‐0002‐TableS2.docx [file BRB3-16-e71353-s002.docx]

**Table S2 Univariate correlation analysis of MMSE scores**

|  | Age | Male | Education | Diabetes | Dyslipidemia | ASCVD history | Smoking | | Exercise | | BMI | HR | SBP | DBP | PP |
| --- | --- | --- | --- | --- | --- | --- | --- | --- | --- | --- | --- | --- | --- | --- | --- |
| r | -0.428 | 0.049 | 0.242 | -0.002 | 0.020 | -0.190 | -0.076 | 0.006 | | 0.060 | | 0.019 | -0.049 | 0.030 | -0.115 |
| *P* | 0.000 | 0.324 | 0.000 | 0.974 | 0.692 | 0.000 | 0.128 | 0.935 | | 0.373 | | 0.781 | 0.325 | 0.548 | 0.020 |

|  | PI | ABI | BaPWV | | TC | TG | FBS | SCr | Antihypertensive drugs | Antidiabetic drugs | Antiplatelet drugs | Stain |
| --- | --- | --- | --- | --- | --- | --- | --- | --- | --- | --- | --- | --- |
| r | -0.132 | 0.380 | -0.122 | -0.034 | | 0.092 | -0.056 | -0.003 | -0.026 | -0.078 | -0.040 | -0.072 |
| *P* | 0.008 | 0.000 | 0.014 | 0.619 | | 0.170 | 0.428 | 0.970 | 0.703 | 0.247 | 0.553 | 0.283 |

MMSE, Mini Mental State Examination; ASCVD, arteriosclerotic cardiovascular disease; BMI, body mass index; HR, heart rate; SBP, systolic blood pressure; DBP, diastolic pressure; PP, pulse pressure; PI, pulse pressure index; ABI, ankle-brachial index; BaPWV, brachial-ankle pulse wave velocity; TC, total cholesterol; TG, triglyceride; FBS, fast blood sugar; SCr, serum creatinine
